# SIgA Binding to Mucosal Surfaces Is Mediated by Mucin-Mucin Interactions

**DOI:** 10.1371/journal.pone.0119677

**Published:** 2015-03-20

**Authors:** Hannah L. Gibbins, Gordon B. Proctor, Gleb E. Yakubov, Stephen Wilson, Guy H. Carpenter

**Affiliations:** 1 Salivary Research Unit, King’s College London Dental Institute, London, United Kingdom; 2 Australian Research Council Centre of Excellence in Plant Cell Walls, School of Chemical Engineering, The University of Queensland, Queensland, Australia; 3 Unilever R&D Discover, Colworth Science Park, Sharnbrook, United Kingdom; Indian Institute of Science, INDIA

## Abstract

The oral mucosal pellicle is a layer of absorbed salivary proteins, including secretory IgA (SIgA), bound onto the surface of oral epithelial cells and is a useful model for all mucosal surfaces. The mechanism by which SIgA concentrates on mucosal surfaces is examined here using a tissue culture model with real saliva. Salivary mucins may initiate the formation of the mucosal pellicle through interactions with membrane-bound mucins on cells. Further protein interactions with mucins may then trigger binding of other pellicle proteins. HT29 colon cell lines, which when treated with methotrexate (HT29-MTX) produce a gel-forming mucin, were used to determine the importance of these mucin-mucin interactions. Binding of SIgA to cells was then compared using whole mouth saliva, parotid (mucin-free) saliva and a source of purified SIgA. Greatest SIgA binding occurred when WMS was incubated with HT29-MTX expressing mucus. Since salivary MUC5B was only able to bind to cells which produced mucus and purified SIgA showed little binding to the same cells we conclude that most SIgA binding to mucosal cells occurs because SIgA forms complexes with salivary mucins which then bind to cells expressing membrane-bound mucins. This work highlights the importance of mucin interactions in the development of the mucosal pellicle.

## Introduction

The mucus layer is essential for protection, molecular transport and lubrication on soft tissues and linings of most of the essential organs. Typically in airways and gastrointestinal tract the mucosal film is formed primarily by mucins, while in other linings like that in the oral cavity the mucosal film (salivary pellicle) also contains globular proteins and proline-rich proteins. Among these globular proteins secretory IgA (SIgA) plays an important role in topical immune response of the adsorbed proteinaceous film. While mucins spontaneously assemble on mucosal surfaces *in vivo*, this behaviour has never yet been fully replicated *in vitro* using purified mucins. The inability to replicate the mucosal layer stems from two key factors. Firstly, purification of proteins leads to the loss of their tertiary conformation, even if mucin preparations are made taking extra care to preserve its gel properties. Secondly, the substrates for *in-vitro* measurements are usually inorganic (or plastic) materials that are significantly dissimilar from the native surface of the cell or connective tissue of the linings. Thus, it has been shown that MUC5B and MUC7 are strongly retained on the buccal cell surfaces, with minimal retention of other salivary proteins [1]. This is in contrast with hydrophobised silicon substrates and hydroxyapatite, where proteins such as statherin and proline-rich proteins (PRPs) are thought to initiate pellicle formation and can be found in abundance within the adsorbed film [2, 3].

In this work we adopted an approach that that tackles both issues associated with studying mucus deposition *in vitro*. Firstly we utilised saliva as a mucin source, since saliva is the only mucosal fluid that has ability to self-assemble onto the surface from the bulk solution. Physiologically, saliva is synthesised away from the epithelium and assembles only upon its excretion from the ducts, where the pellicle forms within minutes of exposure to the oral cavity [4]. This approach ensures that possible effects associated with swelling of mucus gels when extruded from the specialised cells (e.g. goblet cells in the GI) do not influence our results. The use of saliva has its complications associated with multiple components such as amylase, SIgA, carbonic anhydrase VI (CAVI) and cystatin S [5]. Secondly we used cell culture as the test substrate. The HT29 and HT29-MTX cell lines are extremely useful as they provide mucus-depleted or mucus-rich substrates that otherwise are extremely similar if not identical. A similar cell line for oral epithelia does not exist but we believe the mechanisms are universal since the major components (SIgA, mucins etc.,) are common to all mucosal surfaces.

Previous studies of mucin binding to synthetic surfaces suggested hydrophobic interactions are a dominant force that drives mucin adsorption [3, 6–8], with some additional factors related to charges interactions. However, it was also noticed that the adsorption process may rely on other proteins for crosslinking. This was evident for several proteins including PRPs and salivary mucins, particularly MUC5B (unpublished data). MUC5B was only able to bind from UWMS, but not SMSL, and even then in minimal amounts. This could indicate different possible binding interactions: structural changes due to source of the MUC5B [9, 10] altering its binding properties or because parotid saliva (PS) proteins were also required for the integration of salivary MUC5B. For example, acidic PRPs may be involved in TGM crosslinking [2, 11] or in protein complexation [12].

It is possible that mucin adherence and retention may be aided by attachment to MUC1, a membrane bound mucin present on the buccal cell surface, through receptor like actions [13, 14]. Other mucin-like glycoproteins, such as gp230, have been identified as being bound to the mucosa [15], which may also provide an attachment point for mucins. The non-glycosylated regions of mucins have been shown, on hydrophobic surfaces, to contribute in the creation of an anchoring layer on the surface whist the glycosylated region protrudes creating viscoelastic properties [16]. This would be essential for contributing to the lubricious effect [8].

MUC7 may further aid in the pellicle formation by forming complexes with SIgA and lactoferrin, which may improve their incorporation into the pellicle layer [17, 18]. IgA is a key factor in the innate immune protection [19]. The secretory component attached to IgA (SIgA complex) could also aid in its incorporation into the mucosal pellicle and could protect against proteolytic activity [20].

The aim of this study was to better understand the development of the mucosal pellicle and specifically the role of mucins, both bound and free, in the absorption of SIgA to the mucosa. HT29 cells were selected as a model epithelial layer as they can be induced (by methotrexate) to secrete and form a layer of bound mucus containing a gel forming mucin MUC5AC [21]. SIgA binding was studied from UWMS, PS and SIgA alone to understand whether or not salivary (free) mucins are important for SIgA incorporation.

## Materials and Methods

### Cell culture

HT29 and HT29-MTX cell lines (Institut für Pharmazeutische Technologie und Biopharmazie, Marburg, Germany) were cultured in Dulbecco’s modified eagles medium (DMEM) containing 4500 mg/L glucose with 10% fetal bovine serum and 1% penicillin-streptomycin (Sigma-Aldrich, Dorset, UK). Cells were seeded into 6-well plates at 1:10 concentration in each well from 75 cm^2^ flask split when approximately 90% confluent. Cells where grown to 3 time points, day 4 (100% confluence), day 8 and day 16 for experimental procedures. At day 8 HT29-MTX cells have started to produce mucus containing MUC5AC [21], which can be seen visually, and at day 16 they have a comparatively thick layer of this mucus.

### Confocal microscopy

Confocal microscopy on live cells was used to confirm the presence of mucus on cell layers. Images were taken using Leica Confocal imaging software, using FITC and TRITC fluorescence at emissions spectra 488 and 568 respectively. After a control image was taken, 10 μl of melamine formaldehyde particles (0.5 μm diameter) were added which are useful in showing the top layer of mucus by reflection and 10 μl of TRITC 4 (100 mg/ml) was added to visualise cell membranes.

### Saliva collection and cell incubation

Unstimulated whole mouth saliva (UWMS) was collected from one female volunteer, on the morning of each experiment, at least 1 hour after consumption of any food or drink. Parotid saliva (PS) was collected using sweet stimulation whilst using a Lashley cup attached onto the inside of the cheek, covering Stenson’s duct from the parotid gland. Informed written consent was obtained for the volunteer to donate saliva following ethical review of the project by the Brent NRES NHS committee (11/LO/1121). SIgA purified from human colostrum (Sigma, Dorset, UK) was prepared at a concentration of 50 μg/ml in serum free media. SIgA was later prepared at 25 μg/ml and 150 μg/ml corresponding to IgA levels in saliva. 1 ml UWMS, PS and SIgA was then added to cells in triplicate for 20 minute or 1 hour incubation at 37°C, after washing cells in PBS. Saliva or media was then removed, and kept and frozen at −20°C, cells were washed in 0.5 ml PBS and homogenised in 0.5 ml of RIPA buffer (Pierce, Thermo Scientific, Rockford, Illinois, USA) according to manufacturer’s instructions.

### Protein concentration determination

BCA assay kit (Thermo Scientific) was used to measure total protein in all cell homogenates according to manufacturer’s instructions. This ensured equal loading of all cell samples for SDS-PAGE, western blotting and IgA enzyme-linked immunosorbent assay (ELISA).

IgA binding on all cell and saliva samples were measured using an ELISA, as previously described [22]. Rabbit anti-human IgA 1:1000 (Dako, Ely, UK) in carbonate buffer was used to coat the ELISA plates overnight. Three washes in phosphate buffered saline with 1% Tween (PBS-T) were completed. Samples were serially diluted down the plate in duplicate alongside the standard and incubated at 37°C for 1 hour, followed by 3 more PBS-T washes. Detecting antibody rabbit anti-human IgA HRP 1:10000 was used for 1 hour (Dako) followed by 3 final PBS-T washes. Substrate was then added consisting of 20 ml sodium acetate, with 3 μl of H_2_O_2_ and 500 μl of 3,3’, 5, 5’ tetramethylbenzidine (3 mg/ml in dimethyl sulfoxide). The reaction was stopped with 2 M sulphuric acid and absorbance was read at 450 nm using a microplate reader (BioRad, Hemel Hempstead, UK).

### SDS-PAGE

SDS-PAGE was completed on cell homogenates. Equal protein amounts of samples were added to LDS sample buffer (1:4) (Invitrogen, Paisley, UK) and DTT reducing buffer (1:10) (Invitrogen), with water to make up volumes and heated at 100°C for 3 minutes. 15 μl of each sample was applied to each lane on a 4–12% Bis-Tris gel (Invitrogen). Electrophoresis was carried out in MES-SDS running buffer (Invitrogen) according to manufacturer’s instructions. Lanes of buccal cell homogenates and TR146 (oral carcinoma cell line) were run as control comparisons to proteins expected.

### Immunoblotting

After completion of protein separation within the gel, western blotting was completed according to manufacturer’s instructions and used to transfer proteins onto a nitrocellulose membrane. Immunoblotting was used to examine specific proteins of interest including: statherin (1:2000), as described previously [23]), cystatin S (1:2000, R and D Systems, Abingdon, UK), MUC7 (1:100) and MUC5B (1:100) (EU Consortium, gifts of Prof. Dallas Swallow, University College London, UK, as described previously [24]). Membranes were blocked in either TBS with 1% Tween (TTBS) pH 7.6 or TTBS with 2% milk powder (Marvel) added. Membranes were probed with primary antibodies at room temperature for 1 hour (overnight for mucin detection), washed in TTBS for 15 minutes and then followed incubation with the required secondary antibody. A final 15 minute wash in TTBS was completed and then the membrane was incubated with a chemiluminescent substrate, western C (Bio-Rad Laboratories Ltd, Hemel Hempstead, UK), for 5 minutes and then read in the ChemiDoc (Bio-Rad) according to manufacturers instructions.

## Results

### MUC5AC mucus development

**Fig 1:**
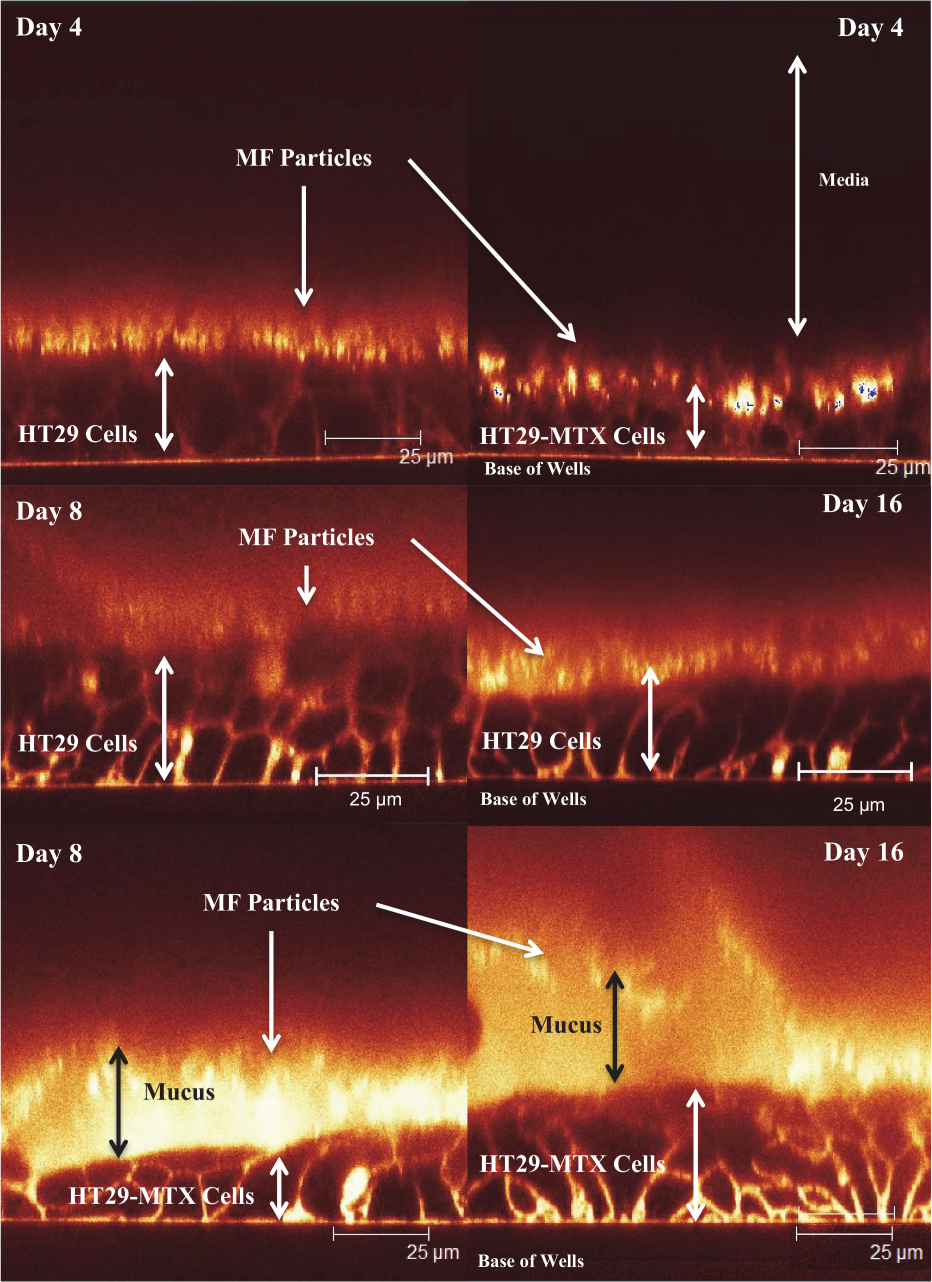
HT29 cell layers at day 4, 8 and 16 and HT29-MTX cells at days 4, 8 and 16, with melamine formaldehyde particles and TRITC 4 added, shows mucus layer on the HT29-MTX only and high lights cell membranes. HT29 cells at day 8 and 16 don’t show any mucus production like the day 4 time point. Arrows are described by labels alongside them, with double-ended arrows highlighting cell and mucus thickness.

Fig. 1 shows CSLM Z-stack cross-sections of the HT29 and HT29-MTX cells at day 4, 8 and 16. The presence of mucus layer was highlighted using reflective MF particles that sat on top of the mucus layer and appeared as bright spots. At day 4 both HT29 and HT29-MTX cells show that MF particles (bright spots) are directly adjacent to the cell plasma membrane surface highlighted by TRITC (which stains the cell membranes). This demonstrates very low mucus production and absence of continuous mucus layer that covers cells. At day 8 and 16 the HT29-MTX cells show the development of a thicker mucus layer that displays continuous coverage. The thickness is somewhat heterogeneous but with no obvious gaps. By contrast, the HT29 cells at day 8 and 16 show very limited mucus production that (when observable) was patchy and thinner compared to HT29-MTX.

**Fig 2:**
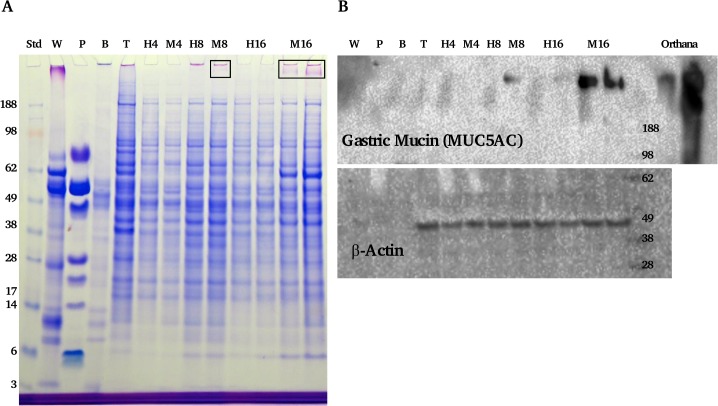
SDS-PAGE gel and wsetern blots of cell homogenates. Panel A shows a gel stained with CBB and PAS of control cells where no saliva or IgA was bound. H indicates HT29 cells with the number indicating the time point, M indicates the HT29-MTX cells. Other lanes include the molecular weight standard, UWMS (W), PS (P), buccal cell homogenate (B) and TR146 cells (T). Boxes highlight mucus band of the MUC5AC. Panel B shows blots of the same samples, probing for MUC5AC and a cell loading control β-actin.

The presence of the mucus layer was further confirmed by gel electrophoresis and western blotting (Fig. 2). Coomassie with PAS staining of cell homogenates revealed the appearance of a high molecular weight glycoprotein band in the MTX cells with time. The blots suggested this band was mostly MUC5AC mucins. It was also evident (although no direct quantification was conducted) that day 16 showed more mucin staining compared to day 8, which is in line with confocal microscopy data.

### IgA binding to cell layers from saliva

**Fig 3:**
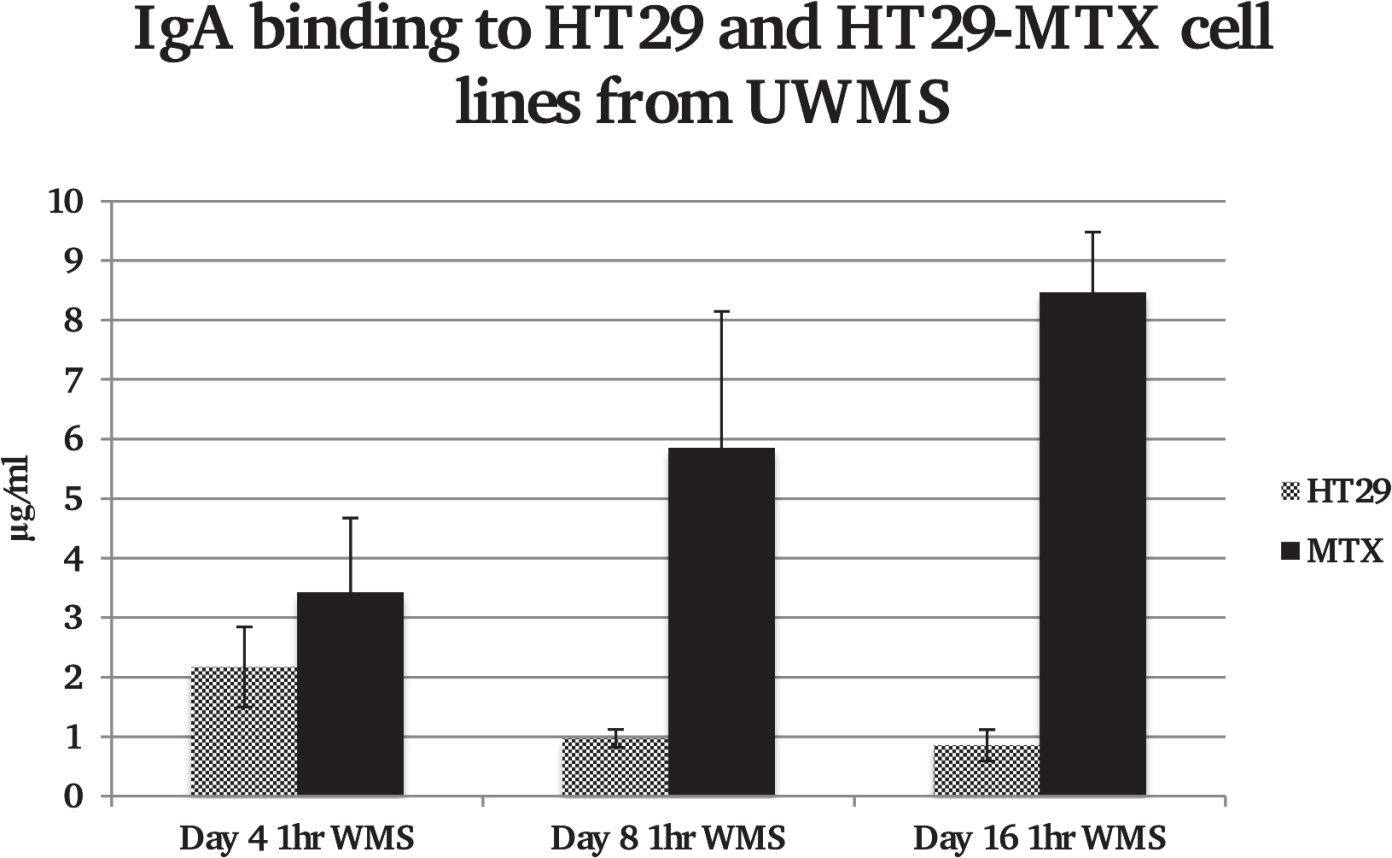
Concentration of IgA bound to equal protein levels of HT29 and HT29-MTX cell lines from UWMS at days 4, 8 and 16. Significantly more IgA is bound to HT29-MTX cells compared to HT29 cells at day 16 and also significantly more compared to the same cell line at day 4, where *P<0.05 Day 16 HT29-MTX compared to day 4 HT29-MTX, **P<0.01 day 16 HT29-MTX compared to Day 16 HT29.

Following a 1 hour incubation of UWMS with both cell lines the amount of IgA bound to the cells was much greater in the presence of the MUC5AC on the HT29-MTX cells at day 8 and day 16, as seen in Fig. 3. At day 16 IgA bound to the HT29-MTX cells was over 8x the amount bound to the HT29 cells (p<0.01). This was significantly more than bound to the HT29-MTX cells at day 4 where there was no MUC5AC mucus development (p<0.05). With a 20 minute incubation with UWMS, at both day 8 and day 16 there was significantly more IgA binding onto the HT29-MTX cells (P<0.05 and P<0.01), in comparison to the HT29 cells without the mucus layer (data not shown).

**Fig 4:**
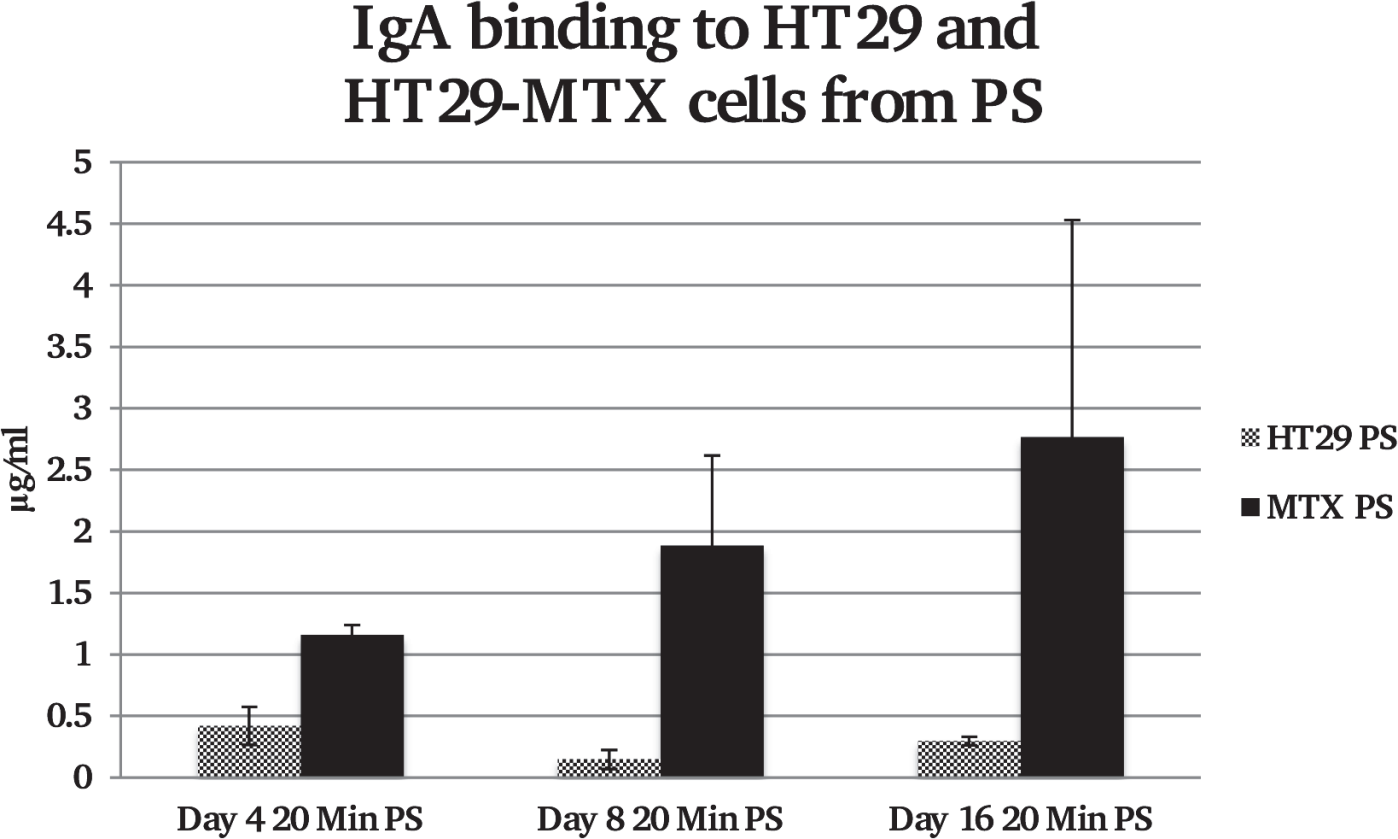
Concentration of IgA bound to equal protein levels of HT29 and HT29-MTX cell lines from PS at days 4, 8 and 16. There does appear to be an increase in IgA binding as the MUC5AC later increases on the HT29-MTX cells but this increase is not significant.

IgA binding from PS also appeared to show an increase in IgA binding onto the HT29-MTX cells (Fig. 4); however a one-way ANOVA suggested there was no significant difference between groups, which is likely to be due to variations of IgA binding to HT29-MTX.

### IgA binding from IgA alone compared to IgA in UWMS

**Fig 5:**
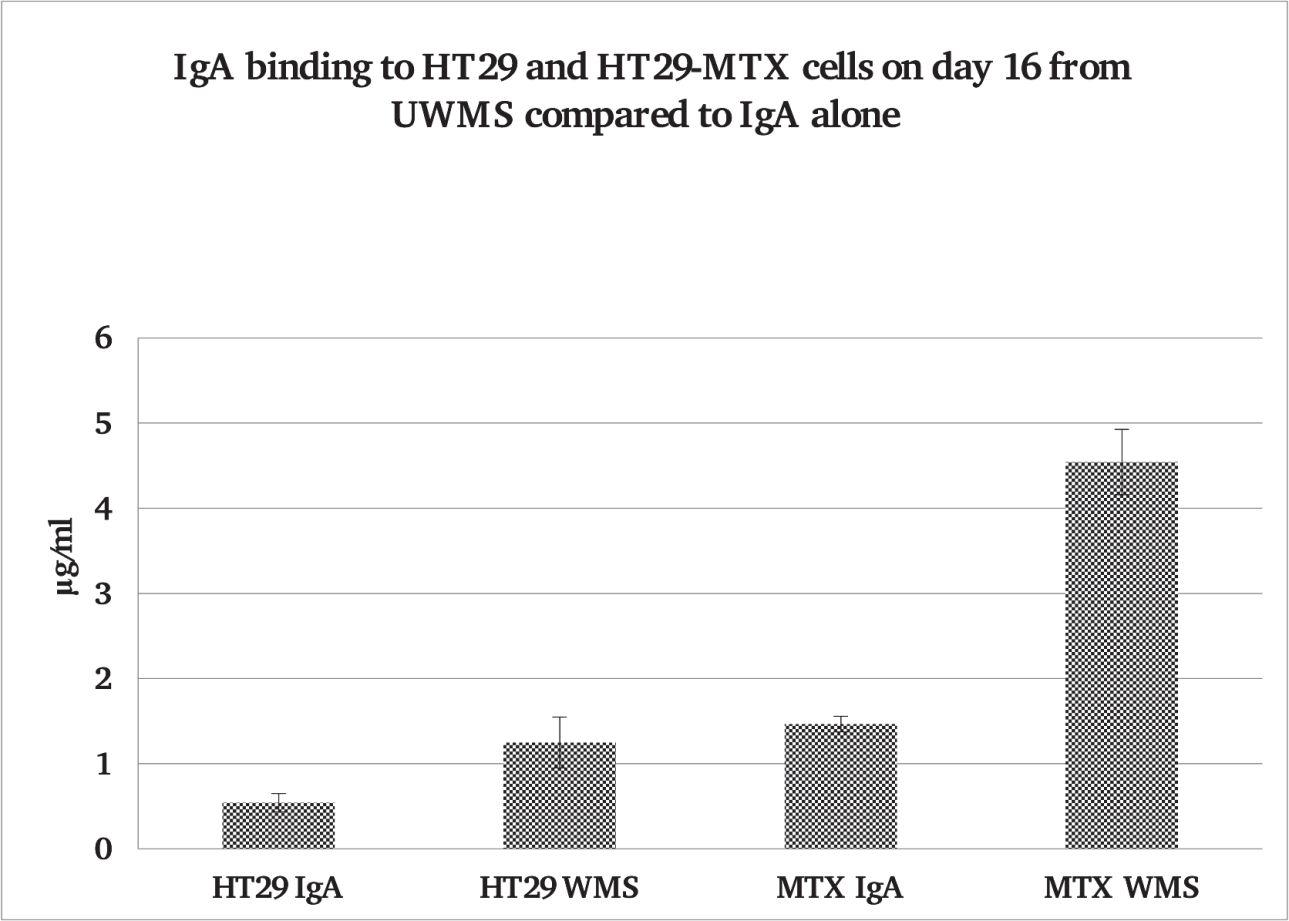
IgA Binding to HT29 and HT29-MTX cells from matched control IgA alone and WMS over 20 minute incubation period. Significantly more IgA bound to HT29-MTX cells from WMS then from IgA, *P<0.01. Significantly more IgA also bound to HT29-MTX then to HT29-MTX cells from IgA alone, *P<0.001.

When a similar concentration of purified SIgA in media was incubated with the HT29 and HT29-MTX cells for 20 minute and compared to UWMS there was significantly less binding compared to the purified SIgA alone (Fig. 5). At day 16, after a 20 minute incubation there was significantly more IgA binding to the HT29-MTX cells from the UWMS (p<0.01). As before there was significantly more IgA binding from UWMS to the HT29-MTX cells, compared with IgA from UWMS binding to the HT29 cells, where MUC5AC was absent from the surface (P<0.001).

When the same experiment was completed with PS, although there appeared to be increased binding from PS to HT29-MTX cells at day 16, this result was not statistically significant.

### Binding of salivary mucins to cell layers

**Fig 6:**
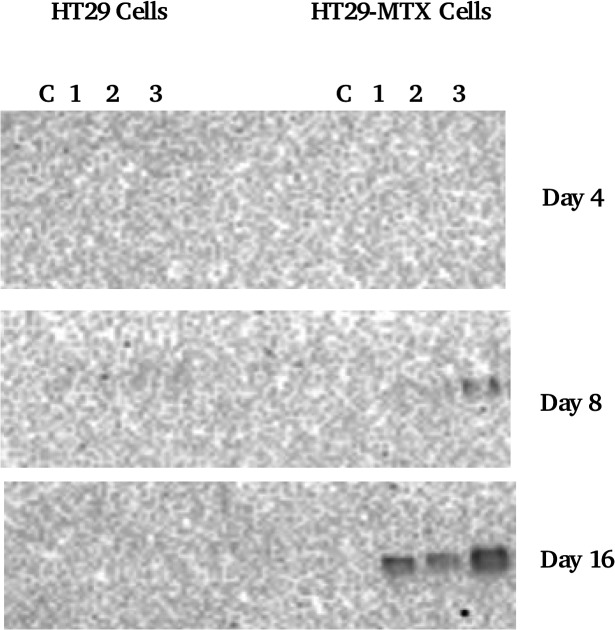
MUC5B western blot of cell homogenates. Lanes 1–3 are cell homogenates where cells were incubated with saliva, C = control (no saliva incubation).

Fig. 6 shows MUC5B binding from UWMS to HT29 and HT29-MTX cells. Salivary MUC5B is one of the two main salivary mucins and it only showed binding to HT29-MTX cells, mostly at day 16 in the presence of the thick mucus layer on the cells, seen in Fig. 3. The other salivary mucin MUC7 showed no binding to either cell lines.

### Binding of other salivary proteins to cell layers

**Fig 7:**
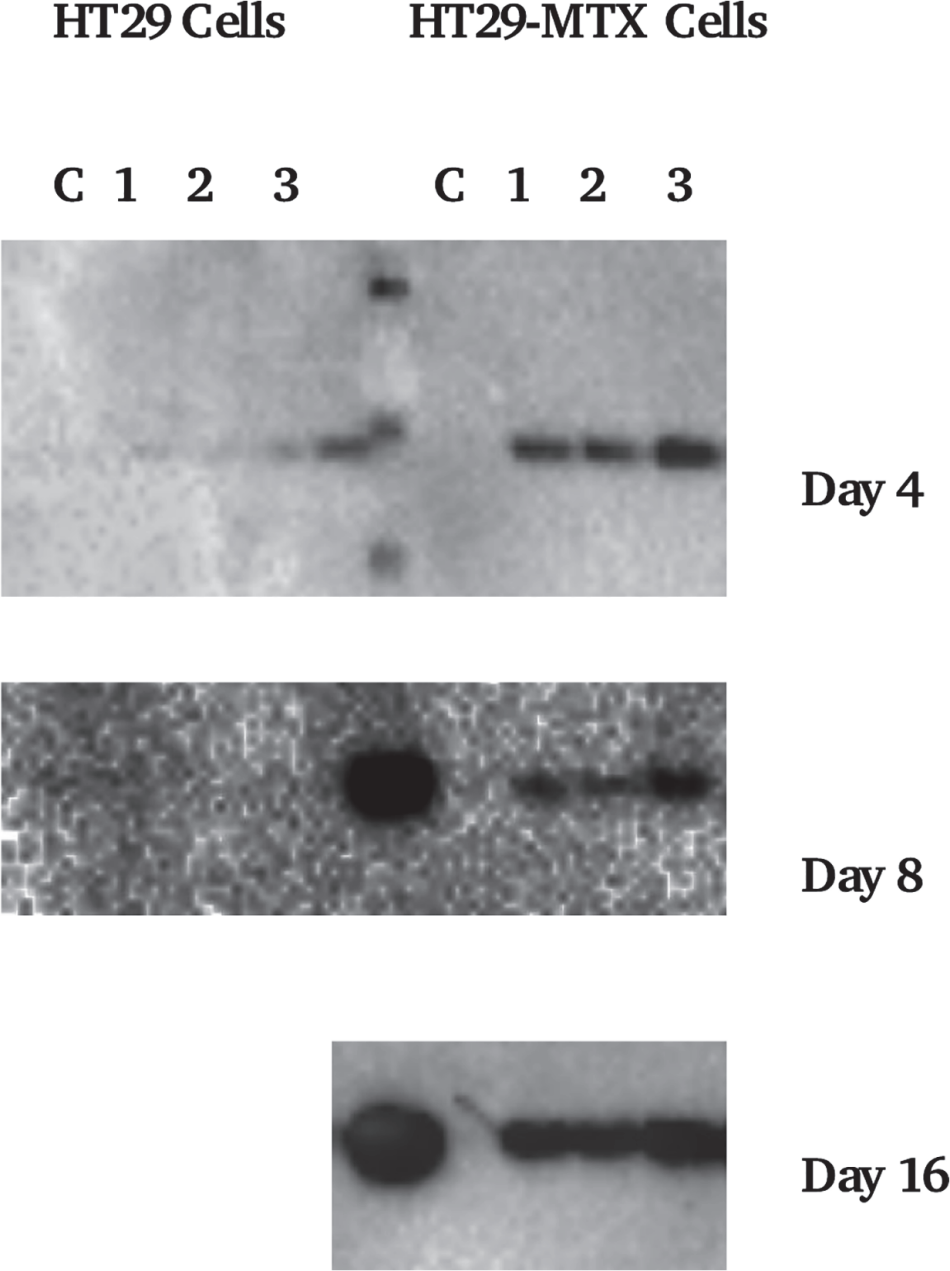
Cystatin S western blot of cell homogenates. Lanes 1–3 are cell homogenates where cells were incubated with saliva, C = control. The centre lane in Day 4 contains a molecular weight standard, whilst day 8 and 16 have WMS added.

Fig. 7 shows cystatin S binding from WMS to HT29 and HT29-MTX cells. Cystatin S was found to bind preferentially to HT29 MTX cells, although by contrast with MUC5B mucin it also binds although to a lesser degree to the HT29 cells. This becomes evident at day 16 where blots showed cystatin S bands for homogenates of both cell lines. Statherin immunoblotting data (not shown) showed no evidence of binding to any cells.

## Discussion

### Mucin binding in the pellicle

The results from this work strongly show the importance of a mucin layer in the development and build-up of a mucosal pellicle. Previously [25] MUC5B was demonstrated to be strongly retained on buccal cells thereby showing its crucial role in pellicle formation on soft oral tissues. Although our previous paper [3] concluded that hydrophobic interactions do drive mucin adsorption, the cell surfaces present an altogether different case. We show that MUC5B deposition is minimal (not detectable by gel electrophoresis and immunoblotting) on the HT29 cells that have no mucus production. The absence of a mucus layer on HT29 cells has been reported previously, and attributed to the fact that the HT29 line contains less than 0.2% of mucus compared to mucus producing goblet cells [26]. It is also known that HT29 cells have suppressed expression of membrane bound MUC1 mucins [27], which otherwise covers nearly all mammalian cells including the oral buccal cells that were tested as being attractive for MUC5B deposition [28]. It is therefore concluded that presence of mucins on the surface of the cells is a key for the formation and further build-up of the mucus layer. The deposited amount correlates with mucin production on the surfaces, as evident from the dependence of mucin and IgA binding on the day in culture. Thus, at day 16 where there was a thick mucus layer of MUC5AC [29] the MUC5B was observed at highest levels, which highlights the importance of mucin-mucin interactions in the development of a protective pellicle [30].

IgA deposition onto cell surfaces from different salivas as well as control IgA solutions also indicated a requirement for mucin-mucin interactions. Time dependent and concentration dependent trends were observed that characterise mucin-assisted IgA binding to the HT29-MTX cells. In Fig. 5 it is clearly shown that adsorption of IgA from control solutions (without mucins) was not statistically different from its deposition on HT29 cells. The deposited amounts were also of the same order as those detected on the HT29 cells following adsorption from the whole saliva samples, suggesting that the level of binding at 1–2 μg/ml marks the level for unspecific binding to the cell surfaces. Only in the case when mucins were present both in solution (as in whole saliva) and on the cell surface (HT29-MTX), were the necessary conditions created for enhanced deposition of IgA to levels of 4.55 μg/ml. The importance of mucins in solution is also evident from data on parotid saliva that lacks mucins. Although there is a trend of increasing IgA deposition with continued culture (and hence with the amount of the surface mucins) this change was not statistically significant due to large variations. The absolute values of deposited IgA are also lower for PS compared with the whole mouth saliva.

In the case of whole mouth saliva, there is a clear concentration dependence (as evident from the time course trend) which allows the deposition of soluble mucins to be attributed to the level of mucus production by the cells. Finally we see that other proteins such as cystatin S that also forms heterotypic complexes with mucins show a similar trend. At the same time statherin was not typically prominent in the pellicle in contrast to those formed on hydroxyapatite surface and air-saliva interfaces [31].

One surprising result was the lack of MUC7 binding, in particular to the HT29-MTX cell or when strong IgA adherence was observed. As described earlier in the paper, MUC7 is known to form complexes with IgA [17] and is also thought to be a strongly bound protein integral to the oral mucosal pellicle [1]. Its lack of binding possibly indicates a lack of interaction with the mucins expressed on the cell lines used in this work. The surface mucins in this cell line are mostly MUC5AC whereas oral epithelial cells tend to express MUC1 and little MUC5AC.

These new insights into mucin binding to mucosal surfaces provide an important step in understanding mucosal pellicle formation as well as help to guide future work to understand pathological conditions such as xerostomia.

## Supporting Information

S1 Data Supporting information—Data Analysis. Excel spread sheets containing raw data for the paper entitled ‘SIgA binding to mucosal surfaces is mediated by mucin-mucin interactions’. Data includes the BCA total protein data for cell homogenates and ELISA results for IgA bound to cells.
(XLSX)
