# Correction: SIgA Binding to Mucosal Surfaces Is Mediated by Mucin-Mucin Interactions

**DOI:** 10.1371/journal.pone.0126887

**Published:** 2015-04-17

**Authors:** The PLOS ONE Staff

There are errors in Fig 7 of the published article. Please see the correct Fig 7 here.

**Fig 7:**
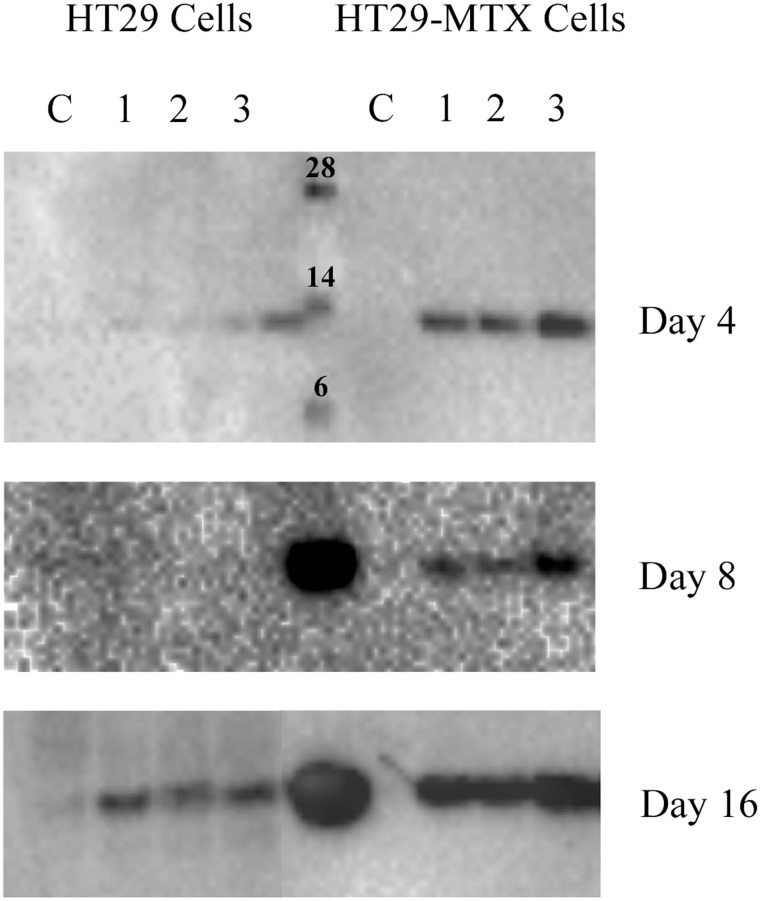
Cystatin S western blot of cell homogenates. Lanes 1–3 are cell homogenates where cells were incubated with saliva, C = control. The centre lane in Day 4 contains a molecular weight standard, whilst day 8 and 16 have WMS added.
